# The possible link between elevated serum levels of epithelial cell-derived neutrophil- activating peptide-78 (ENA-78/CXCL5) and autoimmunity in autistic children

**DOI:** 10.1186/s12993-015-0056-x

**Published:** 2015-03-19

**Authors:** Gehan Ahmed Mostafa, Laila Yousef AL-Ayadhi

**Affiliations:** Department of Pediatrics, Faculty of Medicine, Ain Shams University, Cairo, Egypt; Autism Research and Treatment Center, AL-Amodi Autism Research Chair, Department of Physiology, Faculty of Medicine, King Saud University, Riyadh, Saudi Arabia

**Keywords:** Autism, Autoimmunity, Chemokines, Epithelial cell-derived neutrophil-activating peptide-78, Antineuronal auto-antibodies

## Abstract

**Background:**

In autoimmune disorders, the underlying pathogenic mechanism is the formation of antigen-antibody complexes which trigger an inflammatory response by inducing the infiltration of neutrophils. Epithelial cell-derived neutrophil-activating peptide-78 (ENA-78) is a chemokine that recruits and activates neutrophils, thus it could play a pathogenic role in inflammation and autoimmune disorders. Some autistic children have elevated levels of brain specific auto-antibodies. We are the first to evaluate serum expression of ENA-78 and its relation to antineuronal auto-antibodies in autistic children.

**Methods:**

Serum ENA-78 and antineuronal auto-antibodies were measured by ELISA test in 62 autistic children aged between 4–11 years and 62 health-matched controls.

**Results:**

Serum levels of ENA-78 were significantly higher in autistic children than healthy controls (P < 0.001). Increased serum levels of ENA-78 have been found in 69.35% of autistic patients. In addition, autistic children had significantly higher percent positivity of serum antineuronal auto-antibodies (64.5%) than healthy controls (6.45%), P < 0.001. There was a significant positive association between the positivity of serum antineuronal auto-antibodies and the elevated levels of serum ENA-78 (P < 0.001) in autistic children.

**Conclusions:**

Serum levels of ENA-78 were elevated in autistic children and they were significantly associated with the increased levels of serum antineuronal auto-antibodies. However, these data should be treated with caution until further research is conducted to determine the pathogenic role of ENA-78 in autism and its relation to brain specific auto-antibodies that have been found in some autistic children. The possible therapeutic role of ENA-78 antagonist in autistic children should be also studied.

## Background

Chemokines are a large group of chemotactic cytokines. They have been divided into four groups, designated CXC, CC, C and CX3C, depending on the spacing of conserved cytokines (where X is an amino acid). The CXC chemokines mainly target neutrophils and lymphocytes whereas the CC chemokines target a variety of cell types, including macrophages, eosinophils, basophils and dendritic cells [1]. Recruitment of inflammatory cells plays the key pathogenic role in all inflammatory diseases and autoimmune disorders. Not only are chemokines involved in recruitment of these cells, but they also play a role in their activation and differentiation [2]. Inflammation is characterized by the local tissue expression of chemokines [3-6]. Chemokines and their receptors are important potential therapeutic targets in many inflammatory and autoimmune disorders because of their central role in cell recruitment and activation during inflammation [2].

Epithelial cell-derived neutrophil-activating peptide-78 (ENA-78/CXCL5), is a CXC chemokine that attracts neutrophils [7]. In the healthy tissue, epithelial cells are the primary source of this chemokine. On the other hand, in the inflamed tissue, inflammatory cells that infiltrate the submucosa are the major cellular source of this chemokine [8]. Neutrophils belong to the body’s first line of cellular defense and respond quickly to tissue injury and invading microorganisms [9]. In autoimmune disorders, the underlying pathogenic mechanism is the formation of antigen-antibody complexes, so-called immune complexes (ICs), which trigger an inflammatory response by inducing the infiltration of neutrophils [10].

The presence of auto-antibodies to neural tissues/antigens in autism [11-17] and the increase in the frequency of autoimmune disorders among autistic families [18-21] suggest that autoimmunity may play an important role in the pathogenesis of autism [11]. Chemokines and their receptors have been implicated as functional mediators of immunopathology of autoimmune neuroinflammatory diseases [22,23].

This study was the first to evaluate serum expression of ENA-78 and its relation to antineuronal brain specific auto-antibodies in autistic children.

## Methods

### Study population

This cross-sectional study was conducted on 62 autistic children. They were recruited from the Pediatric Neuropsychiatric Clinic, Faculty of Medicine of Ain Shams University, Cairo, Egypt, during their follow up visits. Patients were fulfilling the criteria of the diagnosis of autism according to the 4^th^ edition of the Diagnostic and Statistical Manual of Mental Disorders [24]. The autistic group comprised 48 males and 14 females. Their ages ranged between 4 and 11 years (mean ± SD = 6.9 ± 1.9 years). Patients who had associated neurological diseases (such as cerebral palsy and tuberous sclerosis), metabolic disorders (eg. Phenylketonuria), allergic manifestations or concomitant infection were excluded form the study.

The control group comprised 62 age- and sex- matched apparently healthy children. They included 47 males and 15 females. They were recruited from the Outpatients Clinic, Children’s Hospital, Faculty of Medicine, Ain Shams University. They were the sibs of the children attending this clinic because of a minor illness (e.g. common cold, tonsillitis and acute bronchitis). The control children were not related to the children with autism, and demonstrated no clinical findings suggestive of infections, allergic manifestations and immunological or neuropsychiatric disorders. Their ages ranged between 5 and 12 years (mean ± SD = 7.08 ± 1.78 years).

The local Ethical Committee of the Faculty of Medicine, Ain Shams University, Cairo, Egypt approved this study. In addition, an informed written consent of participation in the study and its publication was signed by the parents or the legal guardians of the studied subjects.

### Study measurements

#### Clinical evaluation of autistic patients

This was based on clinical history taking from caregivers, clinical examination and neuropsychiatric assessment. In addition, the degree of the disease severity was assessed by using the Childhood Autism Rating Scale (CARS) [25] which rates the child on a scale from one to four in each of fifteen areas (relating to people; emotional response; imitation; body use; object use; listening response; fear or nervousness; verbal communication; non-verbal communication; activity level; level and consistency of intellectual response; adaptation to change; visual response; taste, smell and touch response and general impressions).

*Assessment of cognitive function* (memory, attention, language, concept formation, problem solving, executive and visuospatial functions) with age-appropriate, translated and validated psychometric instruments that were administered by well-trained psychologists using a set of Arabic norms [26] for a translated Wechsler Intelligence Scale for Children, 3rd edition (WISC-III) [27]. This scale is the most commonly used test to assess cognitive function in the children. Three global measures were examined in the present study. The verbal intelligence quotient (IQ) is derived from different subtests including information, similarities, arithmetic, comprehension, vocabulary and digit span. The performance IQ is derived from different subtests including picture completion, block design, picture arrangement, object assembly and digit symbol. The full-scale IQ is the sum of the verbal and performance IQ. The individual subtests may be particularly useful because each depends on a variety of capabilities and dysfunction of any one could result in a low score on one of the global measures. Cognitive dysfunction is diagnosed when the difference between verbal and performance IQ is more than 15 and/or the result of one or more of the individual subtests is below 7 and/or the full scale IQ is below 70.

#### Assessment of serum ENA-78 levels

The assay employs the quantitative sandwich enzyme immunoassay technique (R and D systems Inc., 614 Mclinley Place NE Minenopolis MN 55413, USA). Serum samples were stored at −70°C until assay of ENA-78. A monoclonal antibody specific for ENA-78 has been pre-coated onto a microplate. Standards and samples are pipetted into the wells and any ENA-78 present is bound by the immobilized antibody. After washing away any unbound substances, an enzyme-linked polyclonal antibody specific for ENA-78 is added to the wells. Following a wash to remove any unbound antibody-enzyme reagent, a substrate solution is added to the wells and color develops in proportion to the amount of ENA-78 bound in the initial step. The color development is stopped and the intensity of the color is measured by ELISA recorder. To increase accuracy, all samples were analysed twice in two independent experiments to assess the interassay variations and to ensure reproducibility of the observed results. There were no discordant data between the results (P > 0.05). No significant cross-reactivity or interference was observed.

#### Assessment of serum antineuronal auto-antibodies

This assay employs the indirect immunofluorescence technique (Euroimmun Labaretorium Fur Eperimentelle Immunologie, Germany). Two milliliters of venous blood were collected and transferred into a dry clean tube and left to clot at room temperature. Then, centrifugation was done at 3000 rpm for 5 min. Prompt separation of serum was done. Forzen sections of primate cerebellum covering the reaction area of a biochip slide were incubated with a diluted serum sample. If the sample was positive, specific antibodies of classes IgA, IgG and IgM attached to the neuronal antigens. In a second step the attached antibodies were stained with fluorescence-labeled anti-human antibodies and made visible with the fluorescence microscope. To increase accuracy, all samples were analyzed twice in two independent experiments to assess inter-assay variations and to ensure reproducibility of the observed results (P > 0.05). No significant cross-reactivity or interference was observed.

### Statistical analysis

The results were analyzed by using the commercially available software package (Statview, Abacus concepts, inc., Berkley, CA, USA). The parametric data were presented as mean and standard deviation (SD). In addition, non-parametric data were presented as median and interquartile range (IQR) which is between the 25^th^ and 75^th^ percentiles. Student’s t-test was used for comparison of parametric data, while Mann–Whitney test was used for comparison between non-parametric data. Chi-square test was used for comparison between qualitative variables of the studied groups. Spearman’s rho correlation coefficient “r” was used to determine the relationship between different variables. For all tests, a probability (P) of less than 0.05 was considered significant. The patient was considered to have an elevated serum ENA-78 level if it was above 158.85 pg/ml which was the chosen highest cut-off value (the 95th or the control values as data were non-parametric).

## Results

All studied patients had classic-onset autism. None of the autistic patients had regressive autism, associated neurological diseases (such as cerebral palsy and tuberous sclerosis), metabolic disorders (e.g., Phenylketonuria), allergic manifestations or concomitant infections. The degree of the disease severity was assessed by using CARS and according to this scale, children who have scored 30–36 have mild to moderate autism (n = 28), while those with scores ranging between 37 and 60 points have a severe degree of autism (n = 34). In addition, 34 autistic children had subnormal intellectual function (intelligence quotient below 70); 20 had mild mental retardation (intelligence quotient = 50–69), and 14 had moderate mental retardation (intelligence quotient = 35–49), Table 1. Of the 34 autistic children with subnormal intellectual function, 26 had severe autism and 8 had mild to moderate autism. None of the healthy control children had a neurocognitive disorder.

**Table 1:** **Demographic and laboratory data of children with autism and healthy control children**

		**Children with autism**	**Control group**
		**(n = 62)**	**(n = 62)**
Age (in years):	Range	4-11	5-12
	Mean ± SD	6.9 ± 1.9	7.08 ± 1.78
Sex:	(Male/Female)	48/14	47/15
Intelligence quotient:	Above 70	45.16%	100%
	50-69	32.26%	
	35-49	22.58%	
CARS scores:	Mild to moderate (30–36)	45.16%	
	Severe (37–60)	54.84%	
Serum ENA-78:	Range	110.25-1295.89	60-382.69
(pg/ml)	Median (IQR)	186.26 (105)	112.5 (115)
Positivity of antineuronal antibodies		64.5%	6.45%

CARS, Childhood Autism Rating Scale; ENA-78, epithelial cell-derived neutrophil-activating peptide-78; IQ,R, interquartile ranges.

### Serum levels of ENA-78 in autistic children and healthy control children

**Figure 1:**
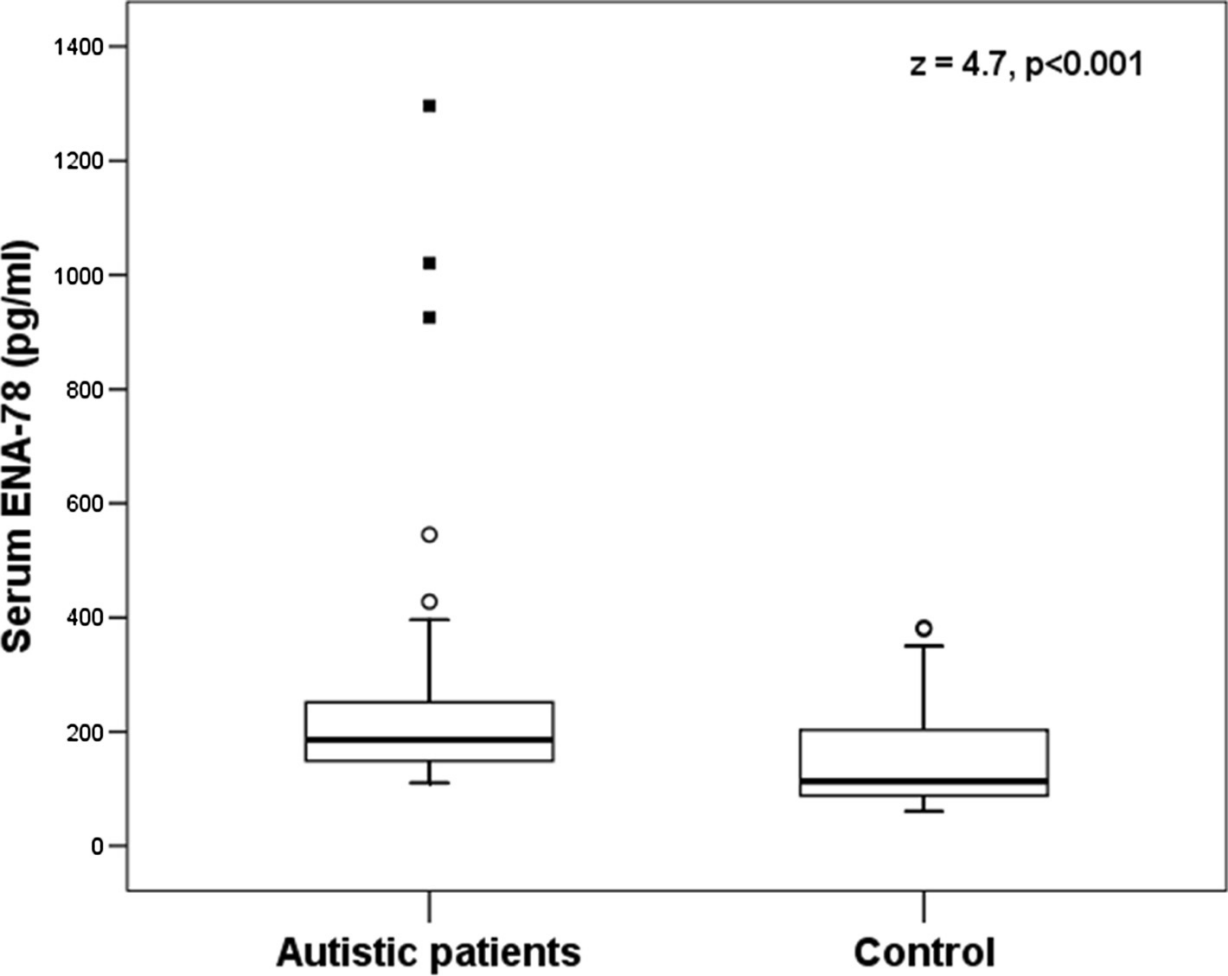
**Serum levels of ENA-78 in autistic patients and healthy controls.** Horizontal bars indicate the median values. ENA-78, epithelial cell-derived neutrophil-activating peptide-78. The boxes enclose the interquartile ranges (IQR) which are between the 25^th^ and 75^th^ percentiles. The horizontal line inside the box represents the median and the whiskers represent the non-outlier (between 1.5 and 3 IQR above the 75th percentile) or extreme (more than 3 IQR above the 75th percentile) maximum and minimum values of serum ENA-78 levels. The small open and black squares represent the outlier (between 1.5 and 3 IQR) or extreme values (more than 3 IQR), respectively of serum ENA-78 levels in autistic children.

Serum levels of ENA-78 were significantly higher in autistic children [median (IQR) = 186.26 (105) pg/ml] than healthy control children [median (IQR) = 112.5 (115) pg/ml], P < 0.001 (Figure 1). Forty three autistic children (69.35%) had increased serum levels of ENA-78.

There were no significant correlations between serum levels of ENA-78 and the age of autistic children, results of CARS and values of intelligence quotient (P > 0.05).

### Positive results of serum antineuronal auto-antibodies in autistic children and healthy control children

Forty autistic children (64.5%) had positive results of serum antineuronal auto-antibodies. Autistic children had significantly higher percent positivity of serum antineuronal antibodies than healthy controls (6.45%; 4/62), P < 0.001.

### The association between increased serum levels of ENA-78 and the positive results of serum antineuronal auto-antibodies

Autistic patients with increased serum levels of ENA-78 had significantly higher frequency of positive results of serum antineuronal auto-antibodies (90%) than patients with normal serum levels of ENA-78 (31.8%), P < 0.001 (Table 2). Thus, there was a significant positive association between the elevated levels of serum ENA-78 and the positivity of serum antineuronal auto-antibodies in autistic children.

**Table 2:** **The frequency of autistic patients with increased serum levels of ENA-78 in relation to elevated levels of serum antineuronal auto-antibodies**

	**Autistic children with elevated serum ENA-78**	**Autistic children with normal serum ENA-78**	**X** ^**2**^ **(p)**
	**(n = 40)**	**(n = 22)**	
Positive antineuronal antibodies (n = 43)	36 (90%)	7 (31.8%)	22.6
Negative antineuronal antibodies (n = 19)	4 (10%)	15 (68.2%)	(<0.001)

ENA-78, epithelial cell-derived neutrophil-activating peptide-78.

## Discussion

Chemokines are a large group of chemotactic cytokines that play an important pathogenic role in inflammatory diseases and autoimmune disorders by enhancement of leukocyte recruitment and activation at inflammatory sites [3-6]. ENA-78 is a CXC chemokine that attracts neutrophils during inflammation [7].

In this work, serum levels of ENA-78 were significantly higher in autistic children than healthy control children (P < 0.001). In addition, 69.35% of autistic children had increased serum levels of ENA-78. This study was the first to investigate serum levels of ENA-78 in autistic children. ENA-78 is an inflammatory C-X-C chemokine that is encoded by the CXCL5 gene [28]. Its levels are elevated in myriad inflammatory conditions [29-32].

ENA-78 is an α chemokine which is produced concomitantly with IL-8 and melanoma growth stimulating activity [7]. The main stimuli for secretion of chemokines, including ENA-78, are the early signals elicited during innate immune response such as bacterial products, viral infection and pro-inflammatory cytokines. Notably, chemokines are induced rapidly, within one hour, by these triggers and provide an important link between early innate immune responses and adaptive immunity [7,8]. ENA-78 can act in concert with IL-8 to stimulate neutrophil directed chemotaxis, neutrophil activation via increasing the intracellular level of free calcium and elastase release and it can also induce neutrophil proadhesive activity [33,34]. Neutrophils contribute to the development of inflammation as they play a role in tissue remodeling. This ability stems in part from the angiogenic property of a subgroup of neutorphil activating CXC chemokines including ENA-78. In the inflamed tissue, infiltrating inflammatory cells are the major cellular source of this chemokine [8]. Immunocytochemistry detected ENA-78 in eosinophils and the peptide was localized in the specific granules. This may suggest that, through expression of ENA-78, eosinophils can recruit and activate CXC receptor 2-bearing cells such as neutrophils at sites of inflammation. Eosinophils may also promote connective tissue remodeling through release of this peptide [7,35].

The transport or synthesis of cytokines in the brain may contribute to neuroinflammation and possible neurotransmitter imbalances in autism [11]. Immunocytochemical studies showed marked activation of microglia and astroglia, and cytokine profiling indicated that macrophage chemoattractant protein-1 and tumor growth factor-beta1, derived from neuroglia, were the most prevalent cytokines in brain tissues. CSF showed a unique pro-inflammatory profile of cytokines, including a marked increase in MCP-1 [36]. Elevated plasma MCP-1, RANTES and eotaxin in some autistic children and their association with more impaired behaviors may have etiological significance [37]. Serum levels of Th2 chemoattractants CCR4 ligands chemokines, macrophage-derived chemokine and thymus and activation- regulated chemokine, were reported to be elevated in autistic children [38]. One study reported elevated serum levels of osteopontin, which is a potent chemo-attractive pro-inflammatory cytokine, produced by immune cells in autistic children [39]. Another study reported reduced levels of progranulin, which is an anti-inflammatory neurotrophic factor, potently inhibit neutrophilic inflammation in autistic children [40]. Chemokines and their receptors might provide unique targets for future therapies in autism [37].

In the current study, 64.5% of autistic children had positive results of serum antineuronal auto-antibodies. A recent study reported positivity of serum antineuronal auto-antibodies in 62.5% of a group of 80 Saudian children with autism [18]. In some autistic children there is an imbalance of T-helper (Th)1/Th2 subsets toward Th2, which are responsible for allergic response and production of antibodies [11]. It is postulated that antineuronal auto-antibodies produce diffuse brain abnormalities and their major autoantigens are unidentified. Antineuronal auto-antibodies bind to the surface membranes of neurons causing direct cytotoxic neuronal injury and affecting the neuronal function [41]. Antineuronal auto-antibodies may play an etiopathogenic role in some autoimmune neurological disorders such as cognitive dysfunction found in patients neuropsychiatric systemic lupus erythematosus and multiple sclerosis [42-44].

Autism may be a disorder of the immune system that occur in a very early phase of embryonic development. Genetic predisposition and environmental predisposition could trigger a deranged immune response which, in turn, results in damage to specific areas of CNS [45]. The pathological role of autoantibodies in development of CNS disorders is a new idea with growing interest among neuroscientists. The involvement of autoimmune response in the pathogenesis of autism has been suggested by the presence of multiple brain-specific autoantibodies in children with autism and in their mothers. The presence of autoantibodies against human neuronal progenitor cells suggest an impaired tolerance to neural antigens in autism. These autoantibodies may affect postnatal neuronal plasticity particularly after impairment of blood–brain barrier [46]. The evidence of a pathomorphic role for 37, 39 and 73 kDa anti-brain antibodies in autism has been recently reported [47]. Immuno-inflammatory pathways and mitochondrial dysfunction play significant interactive roles in driving the early developmental etiology and course of autism [48]. Activation of mast cells in autistic children is accompanied by mitochondrial fission and translocation to the cell surface from where they secrete at least ATP and DNA outside the cell without cell damage. These extracellular mitochondrial components are misconstrued by the body as “innate pathogens” leading to powerful autocrine and paracrine auto-immune/auto-inflammatory responses, a condition that could involve “focal brain allergy/encephalitits” [49].

Despite of the fact that the origin of autoimmunity in autism is unknown, immune related genes on major histocompatibility complex, which have been associated with some autoimmune diseases, may play a central role in the development of autoimmunity in autism such as increased frequency of C4B null alleles in class III region. This results in low production of C4B protein leading to repeated infections which may play an important role in the development of autoimmune reaction to neurons [20,50] through antigenic mimicry, with a subsequent release of neuronal antigens. These neuronal antigens may result in the induction of autoimmune reactions through the activation of the inflammatory cells in genetically susceptible individuals. The formed antibodies may cross the blood brain barrier and combine with brain tissue antigens forming immune complexes, thus further damaging the neurological tissue [11,51].

The current study revealed that autistic patients with increased serum levels of ENA-78 had significantly higher frequency of positive results of serum antineuronal auto-antibodies (90%) than patients with normal serum levels of ENA-78 (31.8%), P < 0.001. Thus, there was a significant positive association between the elevated levels of serum ENA-78 and the positivity of serum antineuronal auto-antibodies in autistic children. This is the first study that investigated the relationship between chemokines and autoimmunity in autism.

Chemokines and their receptors have been implicated as functional mediators of immunopathology of autoimmune neuroinflammatory diseases [22,23,38]. ENA-78 is a CXC chemokine that attracts neutrophils during inflammation [7]. Neutrophils have a pathogenic role in autoimmunity [52]. In autoimmune disorders, the underlying pathogenic mechanism is the formation of antigen-antibody complexes, so-called immune complexes (ICs), which trigger an inflammatory response by inducing the infiltration of neutrophils [10]. The subsequent stimulation of neutrophils by C3b-opsonized ICs results in the generation of reactive oxygen species (ROS) and the release of intracellularly stored proteases leading to tissue damage and inflammation [53]. It is therefore important to identify the mechanisms that control the activation of infiltrating neutrophils [54].

Chemokines and their receptors are important potential therapeutic targets in many inflammatory and autoimmune disorders because of their central role in cell recruitment and activation during inflammation. Targeting the chemokine system is generally done by affecting chemokine receptor binding which can be done in three ways: by using blocking antibodies, by modification of chemokines and therefore antagonizing chemokine receptors and by using small molecule chemokine receptor antagonists [2,55]. The possible therapeutic role of ENA-78 antagonism in autistic children should be studied.

This study revealed that the increase of ENA-78 levels may promote the induction of autoimmunity through stimulation of neutrophil activation. We could not trace data in the literature regarding the neutrophil function in autistic patients, so studies should be conducted to investigate neutrophil function in these patients. As this study is the first that investigated the relationship between chemokines and autoimmunity in autism, we recommend future studies that relate the altered levels of different chemokines and the production of many types of brain specific auto-antibodies in autistic children.

## Conclusions

Serum levels of ENA-78 were elevated in autistic children and they were significantly associated with the increased levels of serum antineuronal auto-antibodies. However, these data should be treated with caution until further research is conducted to determine the pathogenic role of ENA-78 in autism and its relation to brain specific auto-antibodies that found in some autistic children. The possible therapeutic role of ENA-78 antagonism in autistic children should be also studied.

## Abbreviations

CARS: Childhood autism rating scale
EAE: Experimental autoimmune encephalomyelitis
ENA-78: Serum ENA-78
IL: Interleukin
IQR: Interquartile range
MCP-1: Monocyte chemotactic protein-1
ROS: Reactive oxygen species
Th: T-helper cells
